# Maximal strength training in patients with inflammatory rheumatic disease: implications for physical function and quality of life

**DOI:** 10.1007/s00421-022-04948-w

**Published:** 2022-04-19

**Authors:** Håvard Haglo, Ole Kristian Berg, Jan Hoff, Jan Helgerud, Eivind Wang

**Affiliations:** 1 Faculty of Health and Social Sciences, Molde University College, Molde, Norway; 2 Myworkout, Medical Rehabilitation Clinic, Ingvald Ystgaards veg 23, 7047 Trondheim, Norway; 3 Department of Physical Medicine and Rehabilitation, St. Olavs University Hospital, Trondheim, Norway; 4 Department of Circulation and Medical Imaging, Faculty of Medicine and Health Sciences, Norwegian University of Science and Technology, Trondheim, Norway; 5 Department of Medicine, University of Utah, Salt Lake City, UT USA; 6 Department of Østmarka, Division of Mental Health Care, St. Olavs Hospital, Trondheim University Hospital, Trondheim, Norway

**Keywords:** Heavy resistance training, One repetition maximum, Rate of force development, Rehabilitation, Rheumatoid arthritis

## Abstract

**Purpose:**

Patients with inflammatory rheumatic disease (IRD) have attenuated muscle strength in the lower extremities, resulting in impaired physical function and quality of life. Although maximal strength training (MST), applying heavy resistance, is documented to be a potent countermeasure for such attenuation, it is uncertain if it is feasible in IRD given the pain, stiffness, and joint swelling that characterize the population.

**Methods:**

23 patients with IRD (49 ± 13 years; 20 females/3 males), diagnosed with spondyloarthritis, rheumatoid arthritis, or systemic lupus erythematosus, were randomized to MST or a control group (CG). The MST group performed four × four repetitions dynamic leg press two times per week for 10 weeks at ~ 90% of one repetition maximum (1RM). Before and after training 1RM, rate of force development (RFD), and health-related quality of life (HRQoL) were measured.

**Results:**

Session attendance in the MST group was 95%, of which 95% conducted according to MST protocol. Furthermore, MST increased 1RM (29 ± 12%, *p* = 0.001) and early and late phase RFD (33–76%, *p* < 0.05). All improvements were different from the CG (*p* < 0.05). MST also resulted in HRQoL improvements in the dimensions; physical functioning, general health, and vitality (*p* < 0.05). Physical functioning was associated with 1RM (rho = 0.55, *p* < 0.01) and early phase RFD (rho = 0.53–0.71, *p* < 0.01; different from CG *p* < 0.05).

**Conclusions:**

Despite being characterized by pain, stiffness, and joint swelling, patients with IRD appear to tolerate MST well. Given the improvements in 1RM, RFD, and HRQoL MST should be considered as a treatment strategy to counteract attenuated muscle strength, physical function, and HRQoL.

*Trial registration:* ClinicalTrials.gov, NCT04998955, retrospectively registered.

## Introduction

Patients with inflammatory rheumatic disease (IRD), such as rheumatoid arthritis (RA), spondyolarthritis (SpA), and systemic lupus erythematosus (SLE), are characterized by attenuated lower extremities muscle strength (Marcora et al. 2006; Häkkinen et al. 1995; Balsamo et al. 2013). In turn, this attenuation strongly predicts impaired physical function (Andrews et al. 2015), even when controlled for pain, joint swelling, disability score, and disease duration (Madsen and Egsmose 2001). Ultimately, the impaired physical function may lead to reduced health-related quality of life (HRQoL) (Salaffi et al. 2018). Treatments to improve lower extremities muscle strength in patients with IRD, and thus counteract the impaired physical function and reduced HRQoL, are sought after.

Strength training with light to moderate resistance is considered safe as well as beneficial for patients with IRD (Baillet et al. 2012; Sveaas et al. 2017). More than 20 years ago beneficial effects of strength training in patients with rheumatoid arthritis was documented. Increases in dynamic knee extension one repetition maximum (1RM) of 44% following a progressive loading (40–60 to 70–80% of 1RM), 6-month, training intervention was reported, albeit the patients’ rate of force development (RFD) did not exhibit any training-induced improvement (Häkkinen et al. 1997). Although improvements in 1RM undoubtedly are of great importance in this patient population, RFD is an additional and critical measure, since it is recognized to be closely associated with sudden corrective muscle actions (Ochi et al. 2020; Izquierdo et al. 1999) as well as functional performance (Andersen et al. 2010; Aagaard et al. 2002). RFD is also shown to be closely associated with neural factors for force production (Andersen and Aagaard 2006). On this basis, it is certainly interesting that patients with IRD are documented to exhibit a reduction in maximal muscle strength that is considerably larger than what would be expected from the decline in muscle mass alone (Yamada et al. 2017). This indicates that neural factors and muscle intrinsic properties of force production may play an important role in the attenuation of the muscle strength. Indeed, rheumatoid arthritis patients have also been described to have lower RFD compared to healthy controls (Häkkinen et al. 1997).

Strength training that is tailored to not only increase 1RM, but also RFD, through enhancement of neural factors and muscle intrinsic properties, may be an advantageous approach in this patient population. Maximal strength training (MST), utilizing heavy resistance (~ 90% of one repetition maximum; 1RM), and executed with an aim to maximally contract the musculature in the concentric phase of the movement, is indeed such a strength training modality, since it is documented to enhance efferent neural drive to maximally contracting musculature (Tøien et al. 2018) as well as to increase muscle mass (Wang et al. 2017). Of importance, MST is shown to yield almost a twofold increase in 1RM and rate of force development (RFD) compared to conventional strength training consisting of more moderate resistance (70–75% of 1RM) (Heggelund et al. 2013).

Given MSTs great impact on neuromuscular function, and consequently 1RM and RFD, in young (Heggelund et al. 2013; Tøien et al. 2018), old (Wang et al. 2017), and frail patient populations (Mosti et al. 2013; CeŠeiko et al. 2020) MST may have merit as a training modality in patients with IRD. However, the feasibility of utilizing MST in this patient population is dependent on the toleration of the intervention. This is an important query as patients with IRD are characterized by pain, stiffness, joint swelling (Perrotta et al. 2021), and as patients report pain as one decisive disease-related barrier to exercise therapy (Veldhuijzen van Zanten et al. 2015; Fongen et al. 2015). Thus, the aim of this study was to assess if MST (1) was a feasible therapy for patients with IRD, (2) improved 1RM and RFD, and (3) impacted physical function and HRQoL. We hypothesized that 10 weeks of MST in a group of patients with IRD would improve 1RM and RFD, and that this enhanced skeletal muscle force generating capacity would augment the patients’ physical function and HRQoL.

## Methods

### Participants

Twenty-four women and five men with confirmed IRD in the form of RA, SpA or SLE were enrolled into the study through the Norwegian Rheumatic Association, and randomly assigned to MST (*n* = 14) or a control group (CG; *n* = 15). Patient characteristics for individuals that completed the study are given in Table 1. All patients were screened by a medical doctor prior to enrolment. The patients´ IRD had to be stable with no change in disease-modifying antirheumatic drugs over the last 3 months, or anticipated need to change during the intervention. Exclusion criteria were inability to perform the study procedures, pregnancy, unstable ischemic heart disease and planned surgeries influencing study compliance. A maximal oxygen uptake ($\dot{V}$O_2max_) test was used to assess the participants general fitness level, along with measurements of skeletal muscle force generating capacity, and revealed that the participants were relatively sedentary ($\dot{V}$O_2max_, 30.0 ± 5.4 mL·kg^−1^·min^−1^). All participants also reported to be unfamiliar with high intensity strength training. The study was approved by the Regional Committee for Medical and Health Research Ethics in Norway and conducted in accordance with the declaration of Helsinki. All patients reviewed and signed informed consents before participating in the study.

**Table 1:** Descriptive characteristics

	MST (*n* = 13)	CG (*n* = 10)
Female/Male, *n*	11/2	9/1
Age, years, mean (SD)	49 (16)	50 (7)
Height, cm, mean (SD)	170 (6)	170 (4)
BMI (kg‧m^−2^), mean (SD)	27.2 (4.6)	25.8 (3.1)
Diagnosis		
RA, *n*	3	4
SpA, *n*	8	6
SLE, *n*	2	
Disease duration, years, mean (SD)	8 (11)	15 (15)
Medication		
Disease modifying anti-rheumatic drugs, %	77	80
Non-steroidal anti-inflammatory drugs, %	46	40

Data presented as mean (SD) for interpretive and comparative purposes. *MST* maximal strength training, *CG* control group, *BMI* body mass index, *RA* rheumatoid arthritis, *SPA* spondyloarthritis, *SLE* systemic lupus erythematosus

### Study timeline

Both groups were encouraged to keep treatments, nutrition, exercise, and physical activity constant throughout the study period. As a measurement of cardiorespiratory fitness, $\dot{V}$O_2max_ was obtained using an incremental treadmill protocol, on a separate day, before assessment of the patients’ HRQoL and testing of muscle strength. For the latter assessments, a standardized testing protocol was performed 3–5 days before the 10-week study period, and 3–5 days after the last training session carried out by participants in the MST group. All tests were performed in both groups using the same protocols, in the same order, with the same equipment, at the same time of the day, and by the same personnel for the pre- and post-test. The testing started with the participants receiving a HRQoL questionnaire followed by measurements of 1RM and dynamic RFD, respectively, in the lower extremities. Patients were instructed to not perform any intensive activity 48 h before test days.

### Maximal oxygen uptake testing procedures

After warming up for 10 min, the speed was set to 4.5 km h^−1^, and the workload gradually increased by 1.0 km/h or 2% every min. All patients continued their effort until exhaustion, typically within 5–6 min, and encouragement was given to the participants from the tester. Oxygen uptake measurements were measured using a Cortex Metamax II (Cortex Biophysik, Leipzig, Germany) analyzer, and standardized criteria were applied to determine if $\dot{V}$O_2max_ was reached (Wang et al. 2014).

### Strength testing procedures

*Maximal muscle strength:* Following a 10 min warm-up on a treadmill, measurements of 1RM were carried out in a seated horizontal leg press apparatus (Gymleco 343, Sweden). Patients performed a dynamic eccentric–concentric movement initiated from a near 180° angle in the knee joint to 90° in the lowest position, determined visually with assistance of a goniometer, and subsequently back to the starting position after a short (< 1 s) stop. The leg press warm-up procedure consisted of 3 sets with 2–8 repetitions starting with light resistance (50% of estimated 1RM). 1RM was then attained within 3–5 lifts, where the load was progressively increased with 5–10 kg until failure. Patients were given 3–4 min of rest between attempts. The highest load successfully completed was recorded as 1RM. Irrespective of strength training experience or familiarization, the median intraclass correlation coefficient for 1RM has been reported to be good-to-excellent at 0.97 with a low level of variation (Grgic et al. 2020).

*Dynamic rate of force development*: After a 5 min break, dynamic RFD was measured in the same leg press apparatus as used for the maximal strength testing, using a force plate (9286AA, Kistler, Switzerland) mounted on the footplate. External resistance during RFD attempts corresponded to 80% of pretest 1RM, and data was collected at 2000 Hz using Bioware software 2812A1–3 (Kistler, Switzerland). The lifts consisted of a controlled eccentric movement to the 90° knee joint angle, where, after a short (< 1 s) stop, the patients were instructed to perform the concentric movement as fast and forcefully as possible. Three trials were recorded with 3 min rest periods between the trials. The best attempt was used for data analyses. RFD was calculated as Δforce/Δtime within the intervals 0–30, 0–50, 0–100, 0–150, 0–200, and 100–200 ms, where 0 ms represented the commencement of concentric force production. In addition, maximal RFD (RFD_max_) was calculated as the steepest 10 ms of the force–time curve.

### Health-related quality of life

A non-disease dependent self-administered HRQoL questionnaire, Norwegian RAND-36 (Ware and Sherbourne 1992), was given to all patients at pre- and post-test. RAND-36 has been considered to be valid and reliable in the IRD patient population (Linde et al. 2008). The questionnaire evaluates eight dimensions: physical functioning, bodily pain, physical role functioning, general health, vitality, emotional well-being, social functioning, and emotional role functioning. Each dimensions’ outcome is converted to a 0–100 score, where higher scores constitute a better health outcome.

### Maximal strength training

The MST group attended two dynamic leg press sessions per week on non-consecutive days for 10 weeks, supervised by a healthcare professional. All sessions started with two warm-up sets using moderate resistance (~ 40–60% of 1RM), separated by a three min break before commencing four sets of four repetitions with a resistance of ~ 90% of 1RM. A linear progression model was applied, where the load was increased with 5 kg whenever the patients were able to complete more than four repetitions and reduced by 5 kg if they could only perform three repetitions. In addition, a general rule for pain management was implemented. During exercise, some pain was tolerated (≤ 5 on a 0–10 Borg scale) (Sveaas et al. 2020). If the pain exceeded this during the leg press exercise or did not subside within 24 h following training, relevant short-term adjustments were made. Typically, reductions in total training load or decreasing range of motion of the knee joint were applied. Total session training load was modified through lowering resistance with ≤ 20% from previously completed set or reducing the total number of repetitions in a session from 16 to between 8 and 12. Altering knee joint lifting range of motion was done by reducing total range of motion by ≤ 20° in either end of the ~ 180°–90° lifting range.

The training was performed in the same leg press apparatus used during testing and, as in testing, started with a controlled eccentric phase from near 180° to 90° knee joint angle, where the patients were instructed to perform a brief (< 1 s) pause. The brief stop was followed by maximal mobilization of force, to stimulate neuromuscular adaptations (Wang et al. 2017; Tøien et al. 2018), back to a knee angle of near 180°. Including the rest periods of 3–4 min between sets, each session lasted for 15–20 min. The CG did not receive any supervised training during the intervention but were encouraged to keep existing physical activity routines and to follow the Norwegian directory of health’s general advice of physical activity for a minimum of 150 min with moderate intensity, or 75 min with high intensity, or a combination of both, per week. After termination of the study period patients in the CG were given a supervised introduction session to MST.

### Feasibility

Feasibility was evaluated by patient retention, attendance to training sessions and compliance to the MST protocol. Throughout the intervention patients were instructed to report pain or any other discomfort, e.g., fatigue, joint stiffness and/or swelling, that could influence the training (disease related or not) to the healthcare professional supervising the training sessions. Compliance to the prescribed MST protocol was assessed by participants’ individual exercise performance data. In particular, the feasibility of adhering to the progression model. Every session was supervised by a healthcare professional, and all adjustments from each set of the dynamic leg press strength training were recorded in the individual patients’ exercise log and reviewed during the feasibility assessment.

### Statistical analyses

Statistical analyses were blinded for group allocation and performed using SPSS statistics software 26.0 (IBM, Chicago, IL). Figures were made using GraphPad Prism version 8 (GraphPad Software, San Diego, CA). Sample size was estimated based on expected between-group difference in 1RM at post-test. Presuming a standard deviation of 15 kg with an anticipated mean difference of 20 kg between groups, a sample of 18 patients (*n* = 9 in each group) would be required to maintain a statistical power of 0.80 with a two-sided alpha of 0.05. Due to the potential of higher drop-out rates from patient populations we aimed to enrol 30 participants, 15 in each group. Data for the primary outcome measures of force generating capacity exhibited similar Gaussian distribution for both groups. However, due to small sample sizes non-parametric statistics were utilized for significance testing. Within-group differences were analyzed using Wilcoxon signed rank test and Mann–Whitney *U* test was performed to detect between-group differences following the study period. Relationships between changes in variables from pre- to post-test included both the CG and the MST groups and were determined using Spearman rank correlation coefficient. For all tests, level of significance was accepted at *p* < 0.05. For interpretive and comparative purposes data are presented as mean ± standard deviation (SD) in text and tables, and in figures as mean and 95% confidence intervals.

## Results

### Participant compliance and retention

Out of the 29 participants enrolled into the study, 23 patients completed the study within the 10-week intervention period (MST: *n* = 13; CG: *n* = 10). The one patient that withdrew from MST, after 2 weeks, gave inflammation of the ankle joint as the reason for the withdrawal. Although a familiar issue to the patient, the influence of the training intervention cannot be ruled out as a cause for the inflammation. Of the 15 patients randomized to the CG 5 were lost to follow-up. Three of the patients gave medical reasons, unrelated to the experimental procedures, as their reason for withdrawal, while two did not show up for post-testing for unknown reasons. No baseline differences were observed between the two groups and no reported change in medication occurred in either group during the study period.

### Exercise attendance and compliance

The 13 patients in the MST group that completed the study had a mean attendance of 19 ± 1 out of the planned 20 sessions (95%). Of the 243 MST sessions performed altogether, 230 (95%) of them were carried out in accordance with the prescribed protocol. In the continuous feedback to the health professionals supervising MST, none of the 13 patients experienced pain or other disease related discomforts that warranted reporting. Some muscle soreness was reported, however, not more than what is typically expected following this type of strength training. Only the remaining 13 (5%) individual sessions, divided among 7 patients, needed adjustments due to short term pain management. The adjustments entailed one or more of the following: (1) a reduction in exercise resistance with ≤ 10% from the previously completed set, (2) performing less than (but at least half) the prescribed 4 × 4 repetitions, (3) minor alterations in knee joint lifting range of motion. The highest number of sessions needing adjustment for one individual was three. None of the adjustments for any of the 13 sessions were on consecutive sessions or for a repeating issue for any of these 7 patients. Moreover, the adjusted sessions were spread across the duration of the intervention period, specifically, at sessions number: 1, 5, 7, 8, 9, 12, 12, 13, 14, 14, 18, 18 and 20.

### Skeletal muscle force generating capacity

**Fig. 1:**
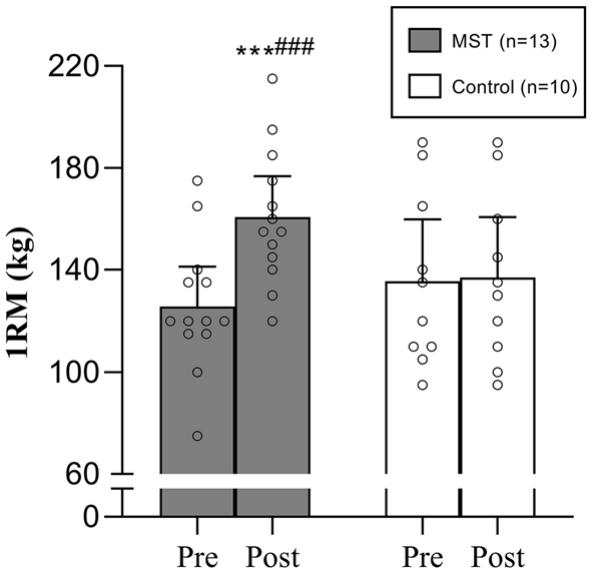
Leg press maximal strength before and after 10 weeks of maximal strength training (MST). 1RM, one repetition maximum. Data presented as mean with 95% confidence intervals and individual responses for interpretation. ***p = 0.001; significant within group difference from pretraining (Wilcoxon signed rank test). ^###^*p* < 0.001; significant difference between groups from pre- to post-test (Mann–Whitney *U* test)

**Fig. 2:**
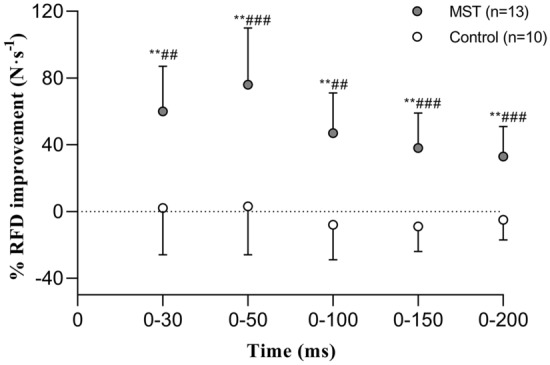
Percentage change improvement in time course of leg press rate of force development (RFD) from pre- to post-training. MST, maximal strength training. Data presented as mean and 95% confidence intervals for interpretation. **p < 0.01; significant within group difference from pretraining (Wilcoxon signed rank test). ^##^*p* < 0.01, ^###^*p* ≤ 0.001; significant difference between groups from pre- to post-test (Mann–Whitney *U* test)

Ten weeks of supervised horizontal leg press training increased the MST groups’ 1RM by 29 ± 12% (*p* = 0.001), with no change occurring in the CG, and this was also apparent as a between-groups difference (*p* < 0.001) (Fig. 1). The MST group improved early phase RFD in the time intervals 0–30 ms, 0–50 ms, 0–100 ms, 0–150 ms by 60 ± 44%, 76 ± 57%, 47 ± 40% and 38 ± 35%, respectively, from pre- to post-training (all *p* < 0.01; Fig. 2). In addition, late phase (100–200 ms) RFD was improved following MST by 35 ± 53% (*p* < 0.05; Table 2). Total RFD (0–200 ms) increased by 33 ± 29% (*p* < 0.01; Fig. 2), while RFD_max_ exhibited a tendency for improvement (20 ± 32%, *p* = 0.06). All RFD improvements were apparent as between-group differences (*p* ≤ 0.001–0.05; Fig. 2; Table 2).

**Table 2:** Leg press rate of force development before and after maximal strength training (MST)

	**MST** (*n* = 13)		**CG** (*n* = 10)	
	Pretraining	Posttraining	Pretraining	Posttraining
0–30 ms	710 ± 338	1067 ± 426**##	900 ± 661	800 ± 447
0–50 ms	945 ± 528	1509 ± 698**###	1150 ± 817	1064 ± 609
0–100 ms	1541 ± 728	2088 ± 657**##	1633 ± 822	1454 ± 749
0–150 ms	1694 ± 584	2236 ± 558**###	1743 ± 613	1549 ± 574
0–200 ms	1578 ± 476	2024 ± 448**###	1593 ± 487	1479 ± 438
100–200 ms	1615 ± 569	1959 ± 528*#	1553 ± 464	1504 ± 490
RFDmax (10 ms)	2885 ± 901	3331 ± 991#	2864 ± 1036	2565 ± 797

Data (in N·s^−1^) presented as mean ± SD for interpretive and comparative purposes. CG, control group. *p < 0.05, **p < 0.01; significant within group difference from pretraining (Wilcoxon signed rank test). ^#^*p* < 0.05, ^##^ *p* < 0.01, ^###^ *p* ≤ 0.001; significant difference between groups from pre- to post-test (Mann–Whitney *U* test)

### Health-related quality of life

**Fig. 3:**
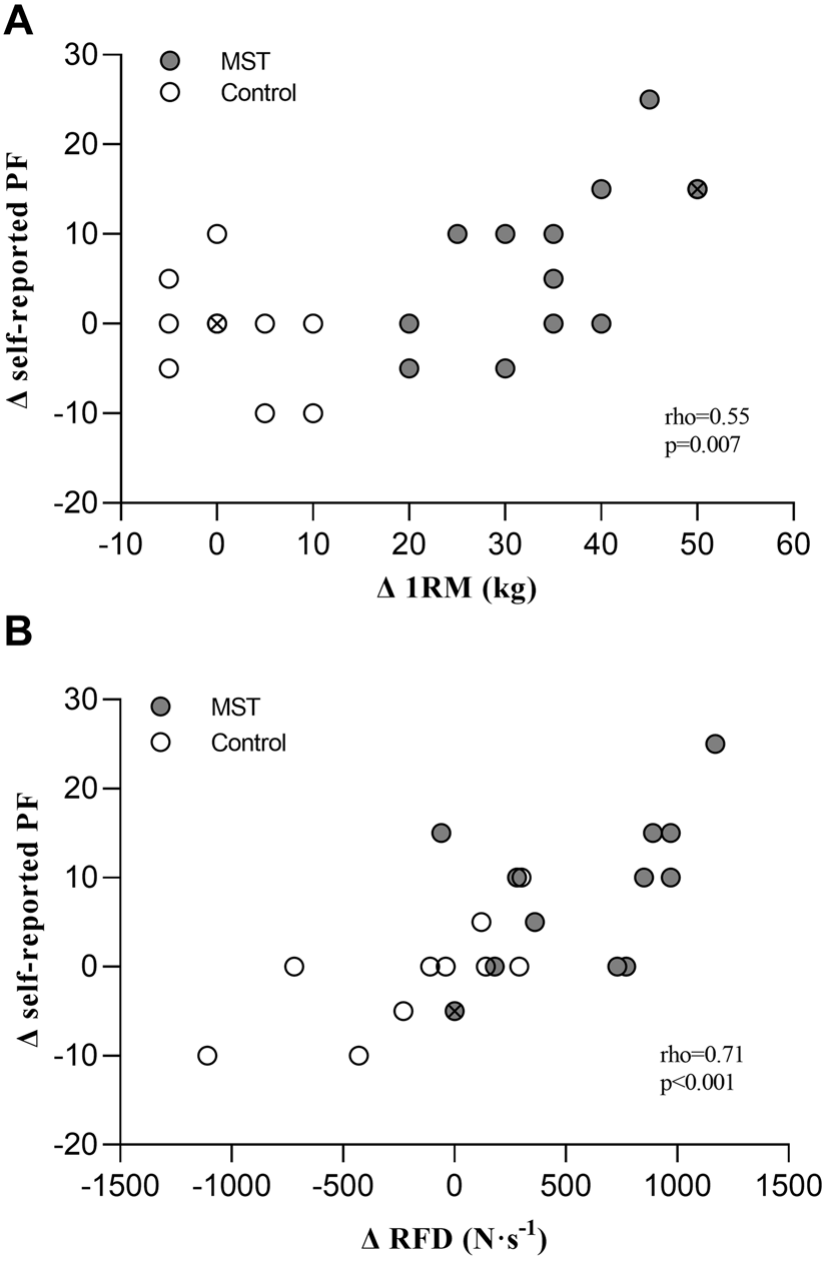
Association (Spearman rank correlation coefficient; rho) between pre- to postintervention differences in the dimension physical functioning (PF) from Rand 36 for (**A**) one repetition maximum (1RM) and (**B**) rate of force development (RFD) 0–100 ms. MST, maximal strength training group (*n* = 13) and Control group (*n* = 10). Data points marked “** × **” indicate two overlapping identical within group values

HRQoL increased in three dimensions following MST. Physical functioning improved by 7.3 ± 9.0 units along with improvements in general health by 9.2 ± 11.9 units, and vitality by 16.5 ± 23.4 units (all *p* < 0.05; Table 3). The minimally clinically important difference in the HRQoL dimensions Physical functioning, general health and vitality have previously been described to be between 3 to 7.7 points, 2.4 to 5 points and 3 to 7.8 points, respectively (Samsa et al. 1999; Kosinski et al. 2000). No changes were detected in the CG following the study period. Between-group differences were observed for the dimensions physical functioning and emotional role functioning (both *p* < 0.05; Table 3). The changes in self-reported physical functioning correlated with changes in 1RM (rho = 0.55, *p* = 0.007; Fig. 3A). Furthermore, associations between changes in physical functioning and changes in RFD in the time intervals 0–30 ms (rho = 0.53, *p* = 0.01), 0–50 ms (rho = 0.62, *p* = 0.002), 0–100 ms (rho = 0.71, *p* < 0.001; Fig. 3B), 0–150 ms (rho = 0.60, *p* = 0.002), 0–200 ms (rho = 0.63, *p* = 0.001), and RFD_max_ (rho = 0.56, *p* = 0.005) were identified.

**Table 3:** Health-related quality of life before and after maximal strength training (MST)

	MST (*n* = 13)		CG (*n* = 10)	
	Pretraining	Posttraining	Pretraining	Posttraining
*RAND 36*				
Physical functioning	77.3 ± 14.4	84.6 ± 14.6*^#^	81.5 ± 11.3	80.5 ± 10.1
Bodily pain	50.4 ± 13.8	55.4 ± 17.8	47.5 ± 12.1	47.5 ± 16.7
Physical role functioning	28.9 ± 43.1	51.9 ± 40.1	40.0 ± 41.2	45.0 ± 43.8
General health	45.8 ± 18.5	55.0 ± 20.4**	49.0 ± 19.0	52.5 ± 17.8
Vitality	43.1 ± 23.2	59.6 ± 17.7*	38.0 ± 16.7	46.5 ± 23.3
Emotional well-being	79.4 ± 10.4	84.0 ± 10.8	76.4 ± 18.6	75.2 ± 16.8
Social functioning	72.1 ± 24.6	80.8 ± 18.1	60.0 ± 25.6	62.5 ± 25.7
Emotional role functioning	82.1 ± 32.2	100.0 ± 0.0^#^	83.3 ± 36.0	66.7 ± 44.5

Data presented as mean ± SD for interpretive and comparative purposes. CG, control group. *p < 0.05, **p < 0.01; significant within group difference from pretraining (Wilcoxon signed rank test). ^#^*p* < 0.05; significant difference between groups from pre- to post-test (Mann–Whitney *U* test)

## Discussion

Patients with IRD suffer from attenuated muscle strength which, in turn, may lead to impaired physical function, and ultimately reduced quality of life (Baillet et al. 2012). Although MST is documented to be an effective countermeasure to this unfavorable outcome, it was unknown whether patients with IRD, who commonly suffer pain, stiffness and joint swelling, could tolerate training with heavy resistance of about 90% of their maximal strength. Thus, the purpose of the current study was to investigate the feasibility and effect of leg press MST in this population. The main findings were that (1) the MST group completed > 95% of the planned MST sessions, of which ~ 95% of completed session were performed in accordance with protocol (2) MST resulted in an increased 1RM, early and late phase RFD (3) The increased force generating capacity was associated with an improved HRQoL related to physical function. No changes were observed in the CG following the study period. Our results reveal that patients with IRD are able to perform strength training with a very high intensity, and that the MST-induced muscle force generating capacity has implications for the patients´ physical function and HRQoL. The results encourage incorporation of MST in future treatment and rehabilitation of patients with IRD.

### MST, IRD, and feasibility

Inflammation is a fundamental clinical sign of rheumatic disease (Schattenkirchner 1987), and symptoms are commonly manifested as musculoskeletal pain, swelling, and stiffness (Sparks 2019). These symptoms could potentially hamper strength training with multi-joint exercises, particularly when applying heavy resistance. The current study documented that ~ 95% of the planned leg press MST sessions were performed in accordance with protocol. This was accomplished without exacerbating any of the HRQoL dimensions, including bodily pain (Table 3) or causing other discomforts affecting the exercise setting.

Increased experience of joint stiffness has previously been reported to act as a barrier to physical activity, while a reduction works as a facilitator (Veldhuijzen van Zanten et al. 2015). Consequently, the high compliance to MST, in combination with the in-session patient dialogue and HRQoL results, indicate a general tolerance to the training regarding these symptoms that frequently impede physical function. In fact, even for the 13 MST sessions that were not performed in accordance with protocol, only minor adjustments were made, and more than half of the sets were carried out as planned. The 13 sessions requiring adjustments were distributed throughout the intervention period, also suggesting that the training did not worsen pain, joint swelling, or stiffness as the 10-week MST intervention progressed.

Considering the very high compliance to protocol, the substantial 1RM improvement of 29% was not surprising, and is in line with similar MST interventions in other patient populations (Wang et al. 2010; Helgerud et al. 2011; CeŠeiko et al. 2020). In fact, the improvement is even somewhat larger than what is observed in healthy individuals across various ages, where an average improvement of 24% has been observed after a total of 24 MST sessions (Kittilsen et al. 2021). This may be due to a larger potential for improvement from a low baseline and/or a mathematically larger percentage increase because of small numbers. The 1.6% 1RM increase per training session in the current study appears to be relatively large compared to previous strength training studies with this patient population. For example, a study by Häkkinen et al. (1997) with inflammatory arthritis patients reported a 1RM increase of 44% following 50 sessions (6 months), implying ~ 0.9% per session.

MST as a strength training method entails a few key factors that may have contributed to its feasibility in this patient population. One important aspect with MST is that the high intensity is limited to the concentric phase of the movement, while the eccentric phase is executed in a controlled and slow fashion, with a short stop before the concentric action. Recognizing that the forces acting on the joints is considerably greater during high-speed eccentric muscle actions compared to concentric (Kellis 2001), MST minimizes the impact of potentially harmful eccentric muscle action (Tøien et al. 2018). A second key feature with MST is the very low volume. The 4 sets are short, rest periods relative long, and each training session is completed in ~ 15–20 min. Considering that the patients only carried out these sessions twice a week in only one exercise, leg press, the overall taxation on the patients´ joints and muscles was very low. A final component with leg press MST, as performed in the current study, is that the repetitions involves a knee joint range of motion from ~ 180° to an angle not lower than 90° in the lowest position, reducing the stress on the hip (Wretenberg et al. 1993) and knee (Cotter et al. 2013) joints that occur below 90° of knee flexion. However, it is uncertain if this reduces the risk of injuries (Hartmann et al. 2013) or pain.

RFD-improvements were also in line with previous MST-induced responses documented in healthy individuals (Tøien et al. 2018; Unhjem et al. 2015; Fimland et al. 2009). In comparison, Tøien et al. (2018) reported, using the same RFD time intervals, increases in the magnitude of 39–62% following leg press MST in healthy young, similar to the 33–76% improvements observed in the present study (Fig. 2). Previously, the early and late phase of RFD is recognized to be affected by different physiological parameters (Andersen and Aagaard 2006). The early phase (< 100 ms) is more influenced by intrinsic contractile properties of the muscle and neural factors (Maffiuletti et al. 2016), while the later phases are more closely related to maximal strength and muscle fiber size (Andersen et al. 2010). However, both phases are dependent on neural factors, with changes in motoneuron firing frequency arguably being the most important (Aagaard et al. 2002). Early and late phase RFD increased following MST in the present study, implying that the participants improved both their qualitative and quantitative neuromuscular properties. Notably, Andersen et al. (2010) suggested that early phase RFD improvements may be caused by the application of very heavy resistance (> 85% of 1RM) in combination with maximal intended velocity. MST involves both principles, this could explain why the patients in the current study exhibited an increase in early phase RFD. In contrast, other studies, where this is not observed, a somewhat lower training resistance without instructions to aim for maximal acceleration has been applied (Narici et al. 1996; Erskine et al. 2014). The increase in early and late phase RFD observed following MST in the current study may have important implications for the patients’ daily physical function and fall prevention (Unhjem et al. 2019), with the early phase RFD indicated to be particularly relevant for very rapid movements (Andersen and Aagaard 2006).

### Inflammatory rheumatic disease, MST, and health-related quality of life

Importantly, following MST in the present study, the increased force generating capacity in the lower extremities resulted in an elevated quality of life. RFD and 1RM both exhibited a strong association with the patients´ reported physical function, with correlations of rho = 0.71 and rho = 0.55 (Fig. 3), respectively. Recognizing that physical function has multifactorial causes, an increase in force generating capacity appears to be one of the factors that may contribute to improved self-perceived physical functioning in patients with IRD. There are several possible everyday scenarios that could explain why the patients in the present study perceived their physical function as improved. First, the relative load of everyday tasks and locomotion would be reduced when force generating capacity is increased, resulting in the experience of a lower taxing of the individual’s capacity in activities of daily living. Indeed, this may be especially germane during more force-dependent daily tasks. As demonstrated by the association of chair rising, and stair climbing to force generating capacity (Unhjem et al. 2019). Second, MST has previously been documented to improve skeletal muscle work efficiency, consequently reducing the oxygen cost of locomotion (Barrett-O'Keefe et al. 2012; Berg et al. 2018). It is likely that MST also increased lower extremities muscle efficiency in the present study, and that this may have contributed to the patients´ perception of elevated physical function when walking. Third, the MST experience, applying very heavy resistance, may also have made patients realize that, despite their disease, it is possible to perform at a near maximal level without worsening their medical condition.

Interestingly, RFD exhibited an even stronger correlation with physical function than 1RM in the current study. This finding underpins the assumption that RFD may be more relevant for physical function than maximal muscle strength (Aagaard et al. 2002; Maffiuletti et al. 2016). As it takes 300 ms or more (Thorstensson et al. 1976) to reach maximal strength, there are many movement types and accelerations that the patients may experience throughout the day, where time is not sufficient to reach maximal strength. Unless patients are highly untrained it is likely few (if any) everyday tasks that require the application of maximal lower extremity strength. Of notice, MST also resulted in an improvement of the patients´ self-perceived general health and vitality, although these observations were not different from the CG. This is in agreement with existing literature indicating the benefits of strength training for physical function and disability (Baillet et al. 2012), while endurance training alone or in combination with strength training appear to be more advantageous for improving pain and vitality (Sveaas et al. 2017). However, the improvements in vitality and general health in this study taken together with the elevated perception of physical function strengthen the assumption with which we can assume that the patients´ overall HRQoL was improved following MST.

### MST and clinical implications

Patients with IRD have attenuated muscle strength and physical function. The current study documents that MST is a potent, time efficient countermeasure to the muscle strength reductions. Moreover, the ability to develop force rapidly is improved, which may be especially relevant, since intrinsic muscle dysfunction has been documented to play an important role in the underlying mechanism of muscle weakness in patients with RA (Yamada et al. 2017). MST has also been documented to increase fast twitch muscle fiber area and percentage in older adults (Wang et al. 2017). Thus, based on the current study and previous literature, MST can be recommended as a safe training modality which targets neural as well as muscular factors and results in improvements in functional performance. Previous studies have also shown that leg press MST may be safely performed in other frail or challenged patient populations, e.g., elderly osteoporotic women (Mosti et al. 2013) or breast cancer patients undergoing chemotherapy (CeŠeiko et al. 2020).

### Study limitations

A larger number of participants and longer intervention period is often sought after in clinical studies. However, as estimated by the power calculation and demonstrated by the results, the intervention duration and number of participants were sufficient to achieve significant difference in the primary outcome measures of muscle strength. A somewhat skewed sex distribution, in favor of females, also occurred in our study. This is not uncommon and may be linked to women being more willing and proactive in choosing health promoting activities (Karstensen et al. 2021; Vervloesem et al. 2012; von Bothmer and Fridlund 2005). Thus, recruitment targeting men may be considered in future studies. In addition, more specific measures of disease activity (e.g., disease specific scores, C-reactive protein, or other biological markers of inflammation) could have added further insights to the feasibility and impact of MST on disease specific outcomes. Advances in pharmacological treatment, and consequently lower symptom burden, may also have contributed to MST tolerance in the current study, and could have been more closely examined. Another limitation to the current study is the lack of direct comparison between MST and other types of strength training. The treatments given by the health care system to the CG were not systematically recorded. Considering individual activity routines and physiotherapy treatments may have varied within the CG it cannot be concluded that MST is more effective than a specific alternative. However, the current design permits comparison of the expected effect of the treatment that is on average offered to patients with IRD. This is a relevant comparison when evaluating MST for implementation in clinical treatment and rehabilitation.

## Conclusions

Although MST is performed with very high intensity and heavy resistance, the results from the current investigation revealed that MST was feasible in patients with IRD and yielded similar increases in force generating capacity as observed in healthy individuals. Importantly, maximal strength, the ability to develop force rapidly and physical functioning was improved without increasing bodily pain. Herein, the early phase RFD, recognized to reflect intrinsic contractile muscle properties, may be of particular importance, since it is documented to be impaired in this patient population. In addition, the patients that performed MST reported enhanced self-perceived general health and vitality. Our results suggest that MST should be considered a potent addition in future treatment and rehabilitation of the patient group.

## Data Availability

Not applicable.

## Abbreviations

CG: Control group
HRQoL: Health-related quality of life
IRD: Inflammatory rheumatic disease
MST: Maximal strength training
RA: Rheumatoid arthritis
RFD: Rate of force development
RM: Repetition maximum
SLE: Systemic lupus erythematosus
SpA: Spondyloarthritis
SD: Standard deviation
SE: Standard error
$\dot{V}$O_2max_: Maximal oxygen uptake
